# Multi-disciplinary management for patients with oligometastases to the brain: results of a 5 year cohort study

**DOI:** 10.1186/1748-717X-8-156

**Published:** 2013-06-27

**Authors:** Jillian Maclean, Naomi Fersht, Mausam Singhera, Paul Mulholland, Orla McKee, Neil Kitchen, Susan C Short

**Affiliations:** 1 Department of Radiotherapy, University College London Hospital, Euston Road, London NW12BU, UK; 2 Department of Medical Oncology, University College London Hospital, Euston Road, London NW12BU, UK; 3 Brain Tumour Unit, National Hospital for Neurology and Neurosurgery, Queen’s Square, London NC1N3BG, UK; 4 Department of Neurosurgery, National Hospital for Neurology and Neurosurgery, Queen’s Square, London NC1N3BG, UK; 5 Leeds Institute of Molecular Medicine, St James University Hospital, Beckett St, Leeds LS97TF, UK

**Keywords:** Brain, Oligometastases, Multidisciplinary specialist clinic

## Abstract

**Background:**

The incidence of oligometastases to the brain in good performance status patients is increasing due to improvements in systemic therapy and MRI screening, but specific management pathways are often lacking.

**Methods:**

We established a multi-disciplinary brain metastases clinic with specific referral guidelines and standard follow-up for good prognosis patients with the view that improving the process of care may improve outcomes. We evaluated patient demographic and outcome data for patients first seen between February 2007 and November 2011.

**Results:**

The clinic was feasible to run and referrals were appropriate. 87% of patients referred received a localised therapy during their treatment course. 114 patients were seen and patient numbers increased during the 5 years that the clinic has been running as relationships between clinicians were developed. Median follow-up for those still alive was 23.1 months (6.1-79.1 months). Primary treatments were: surgery alone 52%, surgery plus whole brain radiotherapy (WBRT) 9%, radiosurgery 14%, WBRT alone 23%, supportive care 2%. 43% received subsequent treatment for brain metastases. 25%, 11% and 15% respectively developed local neurological progression only, new brain metastases only or both. Median overall survival following brain metastases diagnosis was 16.0 months (range 1–79.1 months). Breast (32%) and NSCLC (26%) were the most common primary tumours with median survivals of 26 and 16.9 months respectively (HR 0.6, p=0.07). Overall one year survival was 55% and two year survival 31.5%. 85 patients died of whom 37 (44%) had a neurological death.

**Conclusion:**

Careful patient selection and multi-disciplinary management identifies a subset of patients with oligometastatic brain disease who benefit from aggressive local treatment. A dedicated joint neurosurgical/ neuro-oncology clinic for such patients is feasible and effective. It also offers the opportunity to better define management strategies and further research in this field. Consideration should be given to defining specific management pathways for these patients within general oncology practice.

## Introduction

Brain metastases occur in approximately 20-30% of all cancer patients and have an incidence at least four times higher than primary brain tumours [1]. The common primary sites that metastasise to the brain are lung (45-50%), breast (10-30%), melanoma (5-20%), renal cell (7%) and gastrointestinal tumours (6%). Presentation in the context of an unknown primary also makes up a small proportion of cases (5%) [1-3]. Recent epidemiological data have suggested a very significant increase in brain metastases in patients with common tumour types, primarily related to improved systemic treatment [4].

Historically, brain metastases were considered to be associated with a uniformly poor prognosis and whole brain radiotherapy was the mainstay of treatment. This view has changed gradually in the last two decades with increasing interest in the use of surgery and radiosurgery (RS). Randomised trial evidence now supports the use of surgery and RS in addition to WBRT to improve survival in patients with a single metastasis [5-7]. An increased focus on local treatment for oligometastatic disease is increasingly relevant due to improved systemic therapies in several common cancers, leading to better control of extra-cranial disease and earlier diagnosis of brain metastases due to more widespread use of MRI. In addition, development in radiotherapy technology has made RS more widely available thereby providing a local treatment option for some patients in whom surgery would not be deemed appropriate. An increasing proportion of patients with brain metastases may therefore be candidates for more aggressive local management aimed at optimising local control in brain.

Patient selection for treatment of brain metastases has been addressed by several different approaches to stratification according to prognostic factors [8-10]. The RTOG Prognostic Assessment (RPA) classification is the most widely used and validated. Class 1 and 2 patients are usually considered appropriate for aggressive treatment [11]. Recent publications have suggested additional subdivision based on more detailed information on the status of non-brain metastatic sites and/or tumour biology, which may refine this approach and improve selection further [10,12,13].

Recent randomised studies have added important data on the role of surgery and RS with or without whole brain radiotherapy in good prognosis patients with oligometastases. Data from EORTC 22952–26001 suggest equivalent survival following either modality (10.7 and 10.9 months) and local control rates of 69% and 41% at the treated lesion using RS or surgery respectively [14]. Although local and distant recurrences in the brain were reduced with the addition of WBRT, this did not impact on survival. The authors suggest that with MRI-based follow up, salvage treatment is effective in preventing neurologic progression as a cause of death. Design of this study reflects that fact that many clinicians are moving away from routine use of adjuvant WBRT because of a concern about induction of neurocognitive deficit. Neurocognitive decline in a specific verbal memory domain at 4 months after brain metastases treatment was significantly associated with WBRT in a randomised study [15]. However, very few studies have addressed this issue and several data sets support the view that uncontrolled metastatic disease is a more significant contributor to cognitive problems and poor quality of life [16,17].

In the UK and other countries where there has been increasing site specialisation among oncologists based on primary disease site, there are specific issues in managing patients with brain metastases since they present to their site specialist team, often without links to a neuro-oncology unit. Whilst management outside a neuro-oncology setting may be appropriate for poor prognosis patients, for others lack of access to a comprehensive neuro-oncology opinion may impact on management and outcomes. Lack of specialist management may also limit information that is gathered at follow up and reduce access to new treatment approaches within clinical studies.

Multidisciplinary team working is well established in the management of cancer and joint surgical/ oncology consultations may have a role, particularly where there are several different treatment strategies that have to be balanced against the individual’s circumstances. In view of the changing demographics of metastatic brain disease we established a multi-disciplinary brain metastases clinic in February 2007 specifically for patients with good prognostic features with the hypothesis that improving the process of care could improve clinical management and outcomes in such patients.

## Methods

### Clinic referral criteria

Specific referral guidelines were devised intended to include only patients in RPA classes 1 and 2 suitable for aggressive local management of oligometastases. Patients should meet all criteria: 

•1-5 metastases

•>18 years

•Good performance status (KPS>60)

•Stable or low volume extracranial disease (recent restaging required)

•All pathologies except lymphoma and germ cell tumours

•Location: supratentorial, posterior fossa, skull or skullbase, dural-based or intra-axial

### Clinic logistics

The clinic was held once a fortnight. The same core staff with expertise in neurosurgery, RS and external beam radiotherapy, attended each clinic: a clinical oncologist (neuro-oncology), a neurosurgeon and cancer nurse specialist. Junior staff were not required. The general management principle of the clinic was to offer local therapy (surgery or RS) without WBRT until proven progression after surgery or RS, unless individual circumstances indicated a need for upfront WBRT. Patients were seen in a joint consultation simultaneously by all team members to agree upon a treatment plan. The cancer nurse specialist provided subsequent support to the patients and their families and could involve social services, local palliative care, occupational therapy and vocational rehabilitation as appropriate. If systemic therapies were indicated these would be under the care of the site-specific teams. The same neurosurgeon carried out all elective brain metastases surgery, although all cases were discussed at the neuro-oncology multi-disciplinary meeting (MDM) and emergency cases treated by other neurosurgeons were referred into the brain metastases service via the MDM. Patients being considered for RS were also discussed at a specific RS MDM prior to treatment.

**Figure 1:**
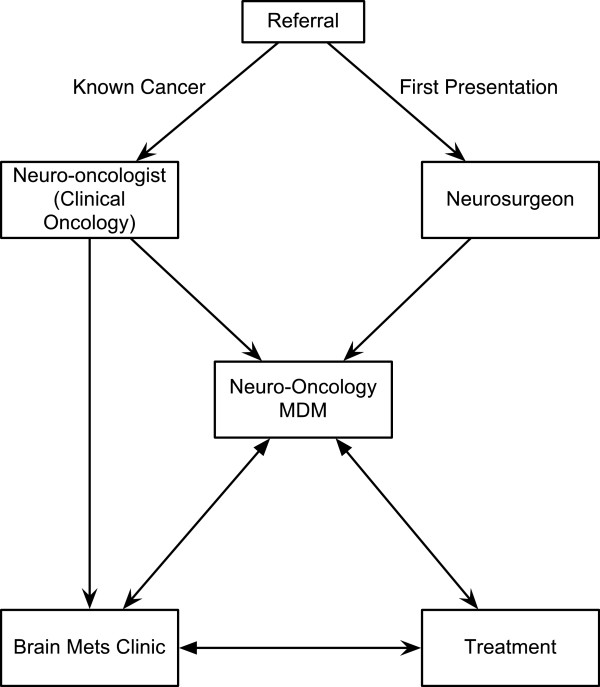
**Management of brain metastases pathway.** The patient continues to see their systemic oncologist for treatment/ monitoring of extracerebral disease as required.

The service was publicised to systemic oncologists through departmental talks within the institution and in nearby cancer centres served by our neurosurgical services. The referral pathway is outlined in Figure 1. In general patients known to have cancer were referred to the clinical neuro-oncologist by their systemic oncologist. Referrals for those at first presentation usually came through the neurosurgical department (referred in from the local hospital, from our own emergency department or hyper-acute stroke unit) and timely referral to the appropriate local site specialist oncologist was then co-ordinated through the metastasis clinic.

Patients were followed up at the brain metastases clinic with contrast enhanced brain MRI scans at 3 monthly intervals, for as long as further aggressive management of any progressive brain disease was deemed appropriate. At the time of progressive brain disease patients were re-evaluated for further local therapy although there was a lower threshold for adjunctive WBRT or partial brain irradiation (including posterior fossa only, or tumour surgical bed). Patients continued to see their systemic oncologist and received systemic therapies as required. Responsibility for patients overall management was retained by their systemic oncologist and they were able to enter any suitable studies for treatment of their systemic cancer.

### Demographic and outcome analysis

We analysed the demographics and outcomes of patients first seen in the clinic between February 2007 and November 2011 to establish whether appropriate patients were being seen and whether patient outcomes supported the role of a dedicated clinic. The analysis was carried out in May 2012 to allow at least 6 months of follow-up per patient. Retrospective data were collected from patient records and imaging held at our institution, other treating hospitals, hospices and the GP. Patient characteristics, treatment modalities, incidence of local brain metastases progression, development of new brain metastases and overall survival with cause of death were analysed. A neurological cause of death was defined as per Patchell et al. [5]. Performance status was recorded in initial clinic documentation using the ECOG scale. Where emergency surgery had been carried out prior to the first clinic appointment performance status was recorded as per the post-surgery clinic visit. To permit RPA classification, ECOG score was converted to Karnofsky Performance Status using the conversion table proposed by Ma [18]. Where patients had been treated elsewhere for brain metastases prior to referral to our clinic, the actual date of brain metastases diagnosis and the initial treatments given elsewhere were recorded in our analysis.

Survival statistics and Kaplan Meier curves were obtained using STATA version 12 and GraphPad Prism version 4. Differences in survival between breast/ lung primary histology and RPA class were assessed for statistical significance using the logrank test. Survival according to treatment modality are stated but differences between treatment groups were not assessed for statistical significance in view of the considerable impact of numerous confounding factors in the non-randomised setting.

## Results

### Clinic activity

114 new patients were managed through the brain metastases clinic (February 2007- November 2011). There were typically 0–2 new patients and 1–3 follow-up patients per clinic in 2007 and 2–3 new patients and 4–6 follow-up patients per clinic in 2012. Overall 25% of referrals involved patients in whom brain metastases were their first diagnosis of cancer and 52% involved the first presentation of brain metastases in patients known to have cancer.18% of patients had initially been treated with WBRT by their systemic oncologist and were referred to the brain metastases clinic when brain progression had developed a considerable period of time after their original WBRT. 46 patients were male and 68 were female with a median age of 59 years (range 22–88 years). 72 patients (63.2%) had a single metastasis at initial presentation, 22 (19.3%) had 2–3 metastases and 20 (17.5%) had ≥4 metastases. Breast and non small cell lung cancer (NSCLC) were the most common primary tumours accounting for 31.6% and 26% respectively. 94 patients (82.5%) had a controlled primary tumour and although 66 patients (57.9%) had extracerebral metastases, generally under control or with effective systemic treatment options available. 106 patients (93%) were ECOG PS 0 or 1. These results corresponded to 26 patients being in RPA class I, 80 patients in class II and 8 patients in class III. Demographic data are summarised in Table 1.

**Table 1:** Patient demographics

**Characteristic**	**n (%)**
**Sex**	
Male	46 (40.4)
Female	68 (59.6)
**Age**	
Mean +/− SD	57.7 +/− 11.8
Median	59.0
Range	22-88
**Primary tumour type**	
Breast	36
NSCLC	29
Melanoma	9
Colorectal	9
Renal Cell	6
Oesophageal	5
Gynaecological	3
Sarcoma	2
Prostate	2
Head and Neck	2
Thyroid	1
Hepatocellular	1
Unknown	9
**Primary tumour controlled**	
Yes	94 (82.5
No	20 (17.5)
**Extracerebral metastases**	
Yes	66 (57.9)
No	48 (42.1)
**ECOG performance status**	
0	59
1	47
2	5
3	3
**Recursive partitioning analysis**	
I	26
II	80
III	8
**Number of metastases**	
1	72 (63.2)
2–3	22 (19.3)
≥4	20 (17.5)

Following treatment over 90% of patients continued scheduled follow up within the brain metastases clinic as planned, a small number who lived further away were monitored by their systemic oncologist only. WBRT was provided by their systemic oncologist if this was more convenient for the patient.

### Treatment

87% of patients received a localised therapy during their treatment course (n=99). Primary treatment was surgery in 70 patients (surgery plus WBRT in 10 patients, surgery plus RS in one patient), RS alone in 16 patients, WBRT alone in 26 patients (including three with boost). Of the patients who received primary WBRT alone, 20 (81%) had received it from their systemic oncologist at least 18 months prior to referral to the brain metastases clinic. Two patients received supportive care only as they were of poor performance status and unlikely to benefit from treatment. 40 patients (35.1%) received a total of two lines of neurological treatment and 9 patients (7.9%) received three or more lines of treatment. Second line treatment was WBRT in 24 patients, RS/ cyberknife (CBK) in 15 patients, surgery in 10 patients (of whom 5 had surgery as first line treatment also) and localised radiotherapy in 2 patients (one post surgery). Subsequent lines of neurological treatment were WBRT in 7 patients or RS/ CBK in 3 patients.

### Outcomes

Data was available for all patients (unknown cause of death for one patient). At the time of analysis, 29 patients were still alive at a median follow-up of 23.1 months (range 6.1 months to 79.1 months).

#### Local control and new brain metastases

Overall, 59 patients (52%) developed neurological progression: 29 patients (25.4%) developed local neurological progression only, 13 patients (11.4%) developed new brain metastases only and 17 patients (14.9%) developed both local progression and new brain metastases. Of the 30 patients who developed new brain metastases distant from the original site, 12 (40%) had received WBRT as part of their primary treatment and 18 (60%) had not. Overall 51 patients (44.7%) did not receive WBRT over the course of their illness. Of the 35 patients who underwent upfront WBRT alone or following surgery, 63% developed neurological disease progression: 10 patients local progression, 9 patients new brain metastases and 3 patients both.

#### Survival

**Figure 2:**
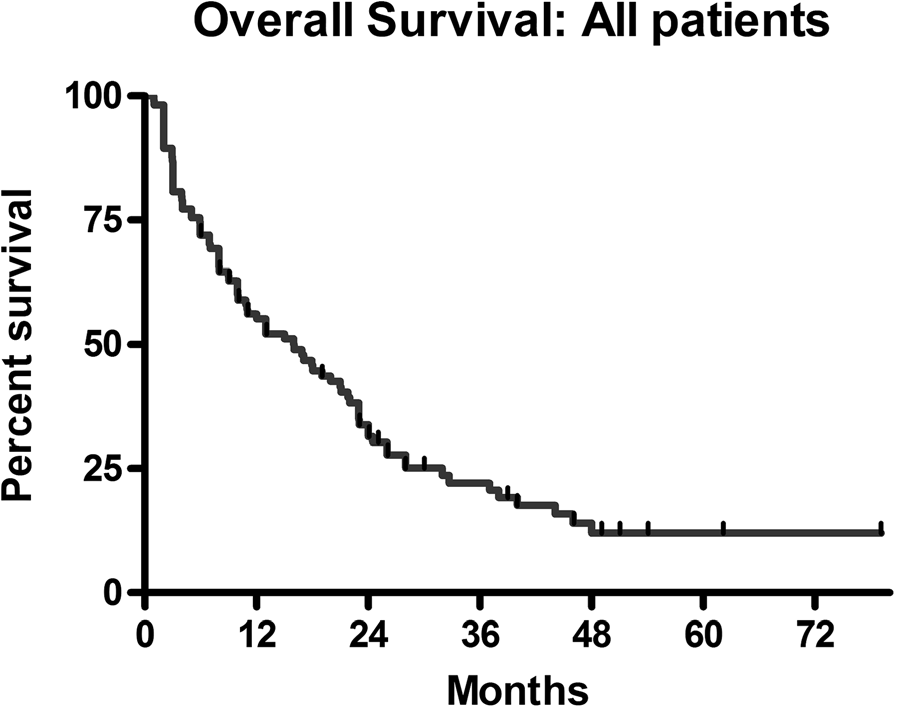
**Overall survival**: **all patients.**

**Figure 3:**
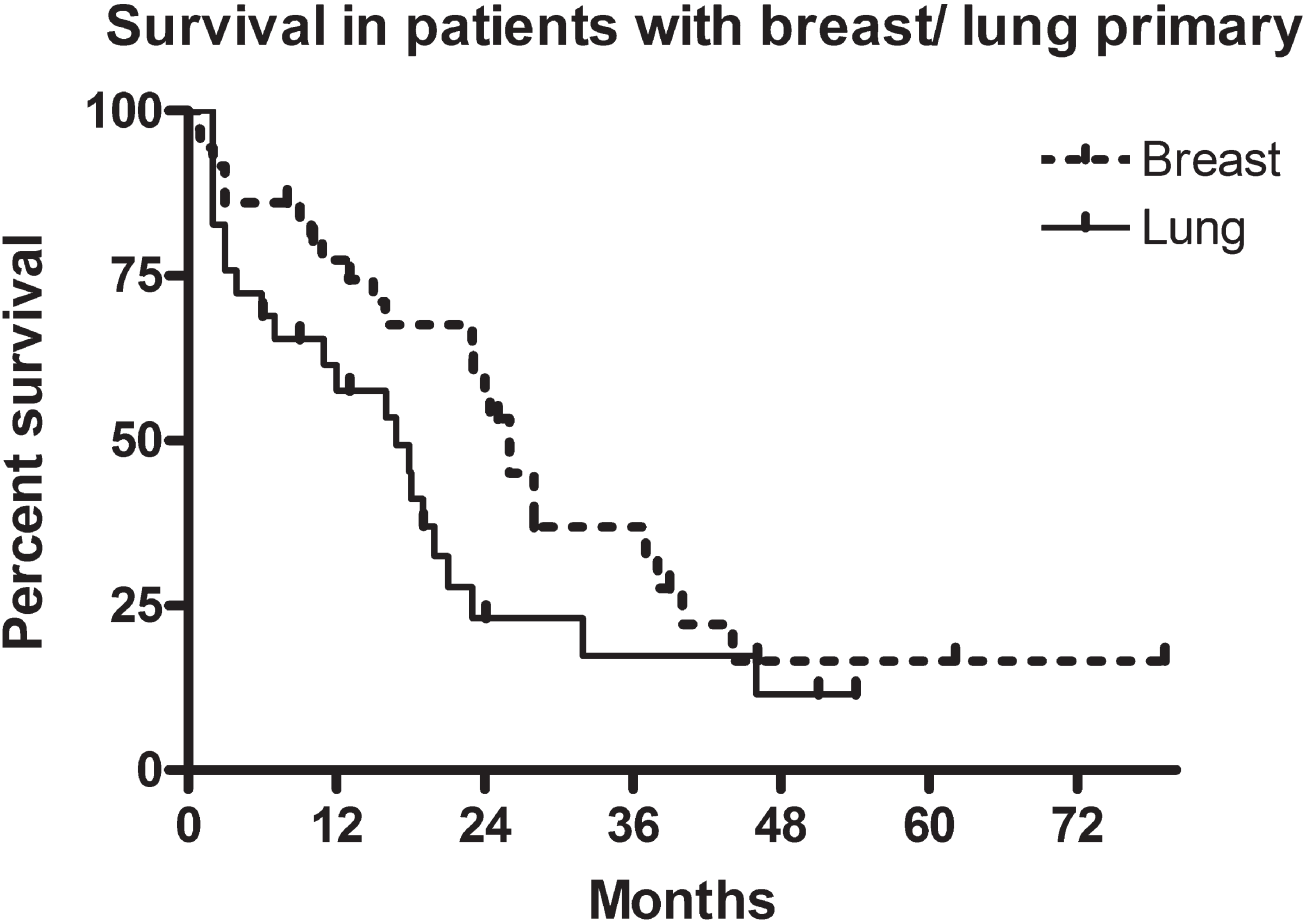
Survival in patients with breast/lung primary.

Median overall survival from diagnosis of brain metastases was 16.0 months across all cancer types (range 1.0-79.1 months) (Figure 2). One year survival was 55% and two year survival 31.5%. When outcomes for the eight patients in RPA class 3 are removed from the analysis, overall median survival was 18 months for those in RPA class 1 and 2 (three months in class 3). There was no statistically significant difference in median survival between those in RPA class 1 and 2 (p=0.76). Median survival in breast cancer patients was 26.0 months and median survival in lung cancer patients 16.9 months, (HR 0.6, p=0.07) (Figure 3). There were 85 deaths in our series, of which 37 (44% of deaths) were classed as neurological deaths.

Table 2 summarises neurological outcomes and survival for the major primary treatment modalities, however, it must be emphasised that confounding factors prevent outcome comparisons and that patients undergoing primary WBRT had already usually survived for over 18 months prior to referral to the clinic hence their apparent longer survival.

**Table 2:** Neurological outcomes

**Primary Tx**	**Patients total (n)**	**1st presentation n (%)**	**Neurological progressive disease** **n (%)**			**Further** **Neuro tx** **n (%)**	**Neuro death** **n (%)**	**Median overall survival (months)**
			**Local**	**New mets**	**Both**			
**Surgery alone**	59	18 (30.5)	12 (20.3)	4 (6.8)	11 (18.6)	29 (49.2)	18 (30.5)	12 (1–79)
**Surgery+ WBRT**	10	6 (60)	4 (40)	1 (10)	1 (10)	4 (40)	2 (20)	16 (8–46)
**RS**	16	1 (6.3)	2 (12.5)	3 (18.8)	1 (6.3)	6 (37.5)	5 (31.3)	13 (2–62)
WBRT *	23	3 (13.0)	7 (30.4)	5 (21.7)	3 (13.0)	10 (43.5)	9 (39.1)	23 (3–46)
**Any**	114	29 (25.4)	29 (25.4)	13 (11.4)	17 (14.9)	50 (43.9)	37 (32.5)	16 (1–79)

*most patients received WBRT from systemic oncologist at least 18 months prior to referral to brain metastases clinic.

## Discussion

Changes in the approach to managing systemic disease in common tumour types associated with brain metastases and the evolving treatment options led us to set up a specific multi-disciplinary clinic to manage good prognosis brain metastases cases with the view that improving the process of care could improve outcomes in this group.

The clinic has proven feasible to run with the same oncologist, neurosurgeon and nurse specialist providing continuity of care. The vast majority of patients seen have met the referral criteria and aggressive local therapy to their brain metastases has been appropriate. Only a small number of patients in RPA class 3 attended the clinic either because they had deteriorated in the time since the referral was made or they had requested a second opinion regarding their management. Patient numbers increased significantly over the last two years which is likely to reflect the time taken to build relationships between site specific teams and the increasing acceptance of locally aggressive management of brain metastases in selected patients within the oncology community. Currently there are approximately 10 patients per clinic and as our referral rate continues to increase we plan to increase to weekly clinics. In view of this continuing increase in referrals it seems likely that not all potentially eligible patients are presently being considered for localised therapy. A regional audit of brain metastases patients would be required to evaluate what fraction of potentially appropriate referrals are seen.

Brain metastases were the first presentation of cancer in 25% and standard site-specific cancer referral pathways do not generally include such patients, particularly where diagnostic uncertainty exists regarding the primary. We have found that managing such patients through the brain metastases clinic has streamlined the organisation of staging investigations and onward referral to the appropriate local site-specific oncologist. Furthermore, we have found that most patients with a new cancer diagnosis find it helpful to receive some general information regarding their cancer and possible further treatment options which is more feasible within our clinic compared to a standard neurosurgical clinic. Support for the patient and their family from the brain metastases specialist nurse in the period prior to seeing the systemic oncologist has proved useful. Concerns regarding the potential of a “metastases-specific” clinic to compromise the decision-making autonomy of systemic oncologists could be seen as a barrier to the development of such services but we are clear to all patients that the systemic oncologist maintains overall responsibility for their care and we have not experienced any conflicts. We have also found that patients are happy to attend both their systemic oncology and brain metastases follow-up clinics. As patients are of good performance status, the extra clinic visit above standard care does not appear to be a particular burden and over 90% attended regular follow-up with us whilst they remained fit enough for further treatment. Good communication between specialists is obviously paramount when disease progression occurs and to ensure that imaging is not duplicated. This was achieved by letter and phone call/ email if appropriate.

This is a deliberately highly selected group, but nevertheless our outcomes are encouraging and represent a fairly typical cancer centre, treating patients referred from routine oncology practice. The 18 month median overall survival of our patients (RPA class 1 or 2) is considerably better than the 7 and 4 months reported in 2000 by Gaspar et al. (RPA class 1 and 2) [11]. These patients had been treated in RTOG 91–04 and patient profiles were similar to ours but localised treatment was not given. This serves to emphasise the importance of localised therapy for brain metastases in appropriate patients and the considerable improvements in the treatment of systemic disease in the last decade. In some common tumour sites effective systemic agents do not reach sites of CNS disease and patients are at risk of neurologic death despite controlled disease elsewhere [19] which underlines the growing need to address CNS disease separately. Without streamlined multidisciplinary management pathways for patients with brain metastases the potential survival benefits of further improvements in systemic therapies may not be realised; with further improvements in survival from systemic metastases more patients are likely to live to develop brain metastases.

There are no trials offering a direct comparison between surgery and RS for oligometastases although available data suggest that local control rates are similar [5,20,21]. A joint surgical/ oncology clinic can reduce clinician bias in treatment decisions in other cancer types [22,23] and we feel that the combined surgical and oncology aspect of our clinic has been beneficial in tailoring treatment to the complex needs of patients with brain metastases. It should be noted in our group that patients treated with primary surgery included patients presenting in emergency settings who may be expected to do less well, whereas those treated with RS were stable pre-treatment.

In the UK, the National Institute for Clinical Excellence (NICE) guidance recommends that referral to neuro-oncology is made for patients in whom brain metastasis is the first presentation of disease or those with a single metastasis and that RS should be available to patients with 1–2 metastases as an alternative to surgery. Until recently RS has been available in only a limited number of centres and there is concern that a substantial proportion of patients have not had ready access to RS. The recent expansion in the number of centres with RS technology should reduce access issues but this underlines the need to identify these patients appropriately [24]. At the same time the availability of RS technologies is encouraging treatment of patients with larger numbers of brain metastases which may define additional sub-groups of patients for whom this is effective treatment, but this is reliant on comprehensive follow-up of patient outcomes [25,26].

The debate continues regarding whether WBRT should be routinely offered following localised therapy for oligometastases to the brain [27]. Data from the EORTC 22952–26001 study have shown that whilst “adjuvant” WBRT was associated with a lower rate of intracranial progression and neurological death, overall survival and functional independence were not improved and that WBRT may negatively impact upon some aspects of quality of life [14,28]. Our policy was to withhold WBRT immediately after localised treatment unless there was evidence of residual disease. 44% of deaths in our study were from neurological causes with the majority of other deaths due to progressive malignancy elsewhere or thromboembolic events, which is consistent with outcomes in the control arm of the EORTC study [14]. 45% of our patients did not require WBRT in their treatment course. Structured MRI follow-up is however essential to identify disease progression in the brain when treatment options were available. The American College of Radiology recommends 3 monthly MRI in the first instance extending to every 4–6 months [29]. Each centre providing a brain metastases service should develop a general policy with regard to WBRT and follow-up.

Although our outcome results are promising, clearly our patient group was highly selected and there are limitations to our analysis. Significant bias prevents and non-randomisation prevents efficacy comparisons between treatments which was not the purpose this report. Due to small numbers, we were unable to calculate survival of less common primary tumours. Quality of life and neuropsychology data was not available, specifically level of functional independence influenced by neurological symptoms. We did not observe significant treatment related toxicity from radiation-based treatments but data on early toxicity following surgery were not available.

Future advantages of treating patients in a specific clinic setting include long-term collection of functional outcome and treatment toxicity. We have now developed a formal database to prospectively collect data and all patients will be offered neuropsychology assessment. The clinic also offers a pathway to support the evaluation of MRI screening in patients at high risk of brain metastases and we will shortly begin evaluating such a policy in high risk patients with metastatic breast cancer (HER-2 positive, hormone negative in the first instance). A dedicated brain metastases clinic should also facilitate trial participation in good prognosis patients and provide a reliable means of documenting long-term outcomes. Emerging research topics in radiation therapy are whether it is the number or volume of metastases that should be used to define patients who likely to benefit from RS, the use of RS to the tumour bed following surgery and the merits of reducing radiotherapy to radiosensitive regions relevant to changes in memory domains, such as the hippocampus [30-32]. Historically patients with brain metastases have been excluded from studies of novel systemic agents, but the advent of small molecule targeted agents that penetrate CNS and the emergence of a good prognosis sub-group of brain metastases patients should change this. Within this group of patients there are very important questions to be addressed including whether novel agents can be selected based on primary tumour molecular genetics and whether the agents that target neo-angiogenesis will be effective in brain metastases.

## Conclusions

The epidemiology of brain metastases is changing. Effective systemic treatments are prolonging survival in common cancers producing a population of “good prognosis” patients with brain metastases who benefit from multi-disciplinary management and rapid access to effective local treatment modalities. We found that a dedicated joint clinic for such patients staffed by a specific interested neurosurgeon, neuro-oncologist and specialist nurse is feasible and effective. We did not experience significant issues with communication/ decision making between systemic oncology and neuro-oncology teams, probably a result of the fact that multidisciplinary teamwork is now standard for many aspects of cancer care. In our experience there is a sufficient need and promising survival data to support a dedicated clinic and continuing patient follow-up in this setting also offers the opportunity to better define management strategies and further research for this patient group. We would encourage other neurosurgical/ oncology centres to develop similar models of care.

## Competing interests

All authors declare that they have no competing interests.

## Authors’ contributions

JM and SS participated in study design, collected and analysed outcome data and drafted the manuscript. NF and NK participated in study design, recorded patient data and drafted the manuscript. MS, PM and OM recorded patient data and drafted the manuscript. All authors approved final manuscript.
